# Rieske Oxygenases: Powerful Models for Understanding Nature’s Orchestration of Electron Transfer and Oxidative Chemistry

**DOI:** 10.1021/acs.biochem.5c00369

**Published:** 2025-08-28

**Authors:** Hui Miao, Sandy Schmidt

**Affiliations:** Department of Chemical and Pharmaceutical Biology, Groningen Research Institute of Pharmacy, 3647 University of Groningen, Antonius Deusinglaan 1, Groningen 9713AV, Netherlands

## Abstract

Rieske oxygenases (ROs) are a diverse family of nonheme iron enzymes that catalyze a wide array of oxidative transformations in both catabolic and biosynthetic pathways. Their catalytic repertoire spans dioxygenation, monooxygenation, oxidative *N*- and *O*-dealkylation, desaturation, sulfoxidation, C–C bond formation, *N*-oxygenation, and C–N bond cleavage—reactions that are often challenging to achieve selectively through synthetic methods. These diverse functions highlight the increasing importance of ROs in natural product biosynthesis and establish them as promising candidates for biocatalytic applications. Despite extensive study, our understanding of how ROs orchestrate these diverse reactions at the molecular level remains incomplete. In particular, the transient, dynamic nature of electron transfer events and the limited structural characterization of oxygen-bound intermediates hinder our understanding of how structural features govern electron transfer efficiency, O_2_ activation, and the origins of their catalytic diversity. Recent findings challenge traditional views of the RO catalytic cycle and underscore the importance of integrating static structural data with dynamic studies of redox interactions. In this Perspective, we explore emerging insights into the structural and mechanistic basis of RO function. We focus on how the architecture of the oxygenase component shapes reactivity, electron transfer, and redox partner interactions. Finally, we discuss current limitations and future opportunities in harnessing ROs for biocatalysis, emphasizing the potential of engineering approaches—particularly the optimization of redox partner compatibility—to expand their functional utility.

## Graphical abstract

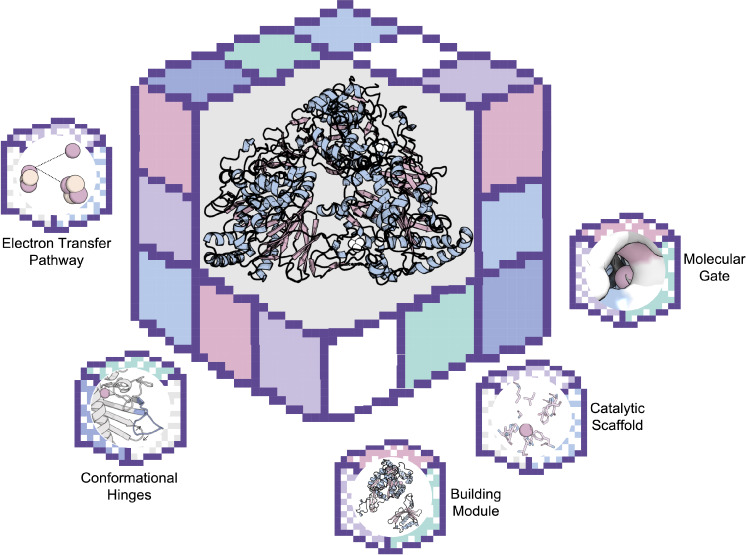

## Introduction

Rieske oxygenases (ROs) are a family of nonheme iron enzymes that catalyze stereo- and regiospecific oxygenation reactions using molecular oxygen.^1^ Predominantly found in bacteria, these enzymes play a crucial role in the catabolism of aromatic hydrocarbons, initiating the degradation of complex organic molecules through hydroxylation.^2,3^ This transformation enables microbial species to access and metabolize aromatic compounds as sources of carbon and energy.^4^ In addition to hydroxylation, ROs exhibit a remarkably broad catalytic repertoire, including oxidative *N*- and *O*-dealkylation,^5–7^ desaturation,^8^ sulfoxidation,^9^ C–C bond formation,^10,11^ *N*-oxygenation,^12^ and C–N bond cleavage (Figure 1).^13,14^ This functional diversity underpins their growing significance in natural product biosynthesis and highlights their potential as versatile tools for green chemistry,^15^ bioremediation,^16^ and the sustainable production of pharmaceutically and industrially relevant compounds.^17^

**Figure 1:**
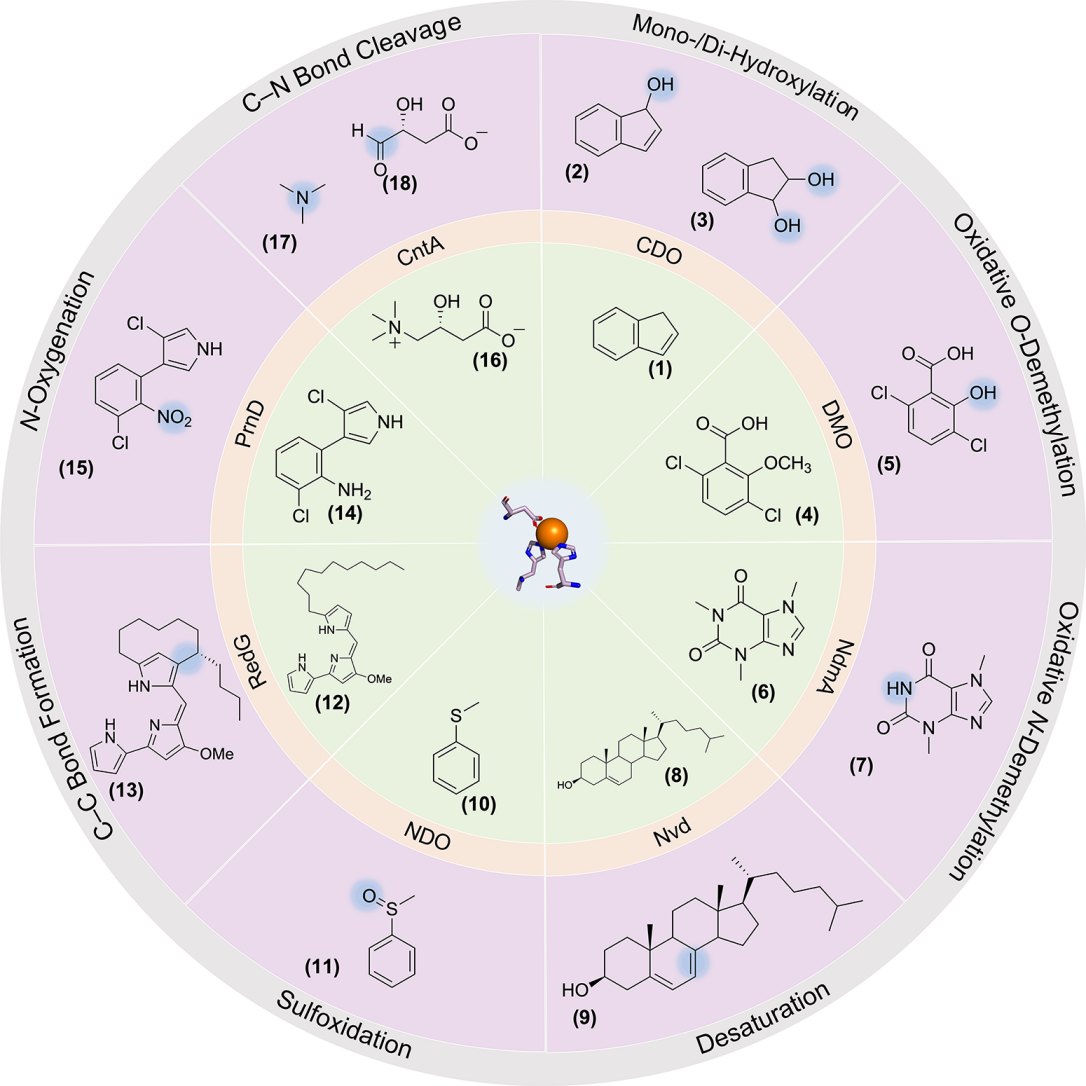
Overview of the Rieske oxygenase reactivity. Mono/dihydroxylation of indene (**1**), catalyzed by cumene dioxygenase (CDO), to form 1*H*-indenol (**2**) and 1,2-indandiol (**3**).^21^ Oxidative *O*-demethylation of dicamba (**4**), catalyzed by DMO, to produce 3,6-dichlorosalicylic acid (**5**).^7^ Oxidative *N*-demethylation of caffeine (**6**), catalyzed by *N*-demethylase A (NdmA), to form theobromine (**7**).^5^ Desaturation of cholesterol (**8**) to afford 7-dehydrocholesterol (**9**), catalyzed by Rieske oxygenase DAF-36/Neverland (Nvd).^8^ Sulfoxidation of thioanisole (**10**), catalyzed by naphthalene dioxygenase (NDO), to afford methyl phenyl sulfoxide (**11**).^9^ Oxidative carbocyclizations of undecylprodigiosin (**12**), catalyzed by RedG, to form streptorubin B (**13**).^10^ *N*-Oxygenation of aminopyrrolnitrin (**14**), catalyzed by aminopyrrolnitrin oxygenase (PrnD), to produce pyrrolnitrin (**15**).^12^ C–N bond cleavage of carnitine (**16**), catalyzed by carnitine oxygenase (CntA), to afford trimethylamine (**17**) and malic semialdehyde (**18**).^13^ Adapted with permission from Papadopoulou et al.^22^ Copyright 2022 American Chemical Society.

The catalytic cycle of ROs is driven by a multistep electron transfer pathway required to activate molecular oxygen, one of the most thermodynamically stable molecules.^18,19^ Electrons are typically supplied by a flavin-containing reductase and transferred via a ferredoxin to the Rieske [2Fe–2S] cluster of the terminal oxygenase, and ultimately to the mononuclear nonheme iron at the oxygenase active site.^1^ Depending on the class, the reductase itself may also contain an iron–sulfur cluster in addition to a flavin. The electron flow is essential for generating the reactive Fe-oxygen species needed for substrate oxidation and represents a key feature of their enzymatic mechanism.^20^

Despite decades of investigating key aspects of RO catalysis—such as how structural elements within oxygenases govern electron transfer efficiency and directionality, how O_2_ is activated, and what causes the broad diversity of catalytic activity observed across RO families—it is still unclear how enzymatic reactions are triggered within these complex systems. Limited structural resolution of oxygen-bound intermediates and the transient, dynamic nature of electron transfer events have restricted direct mechanistic insight. Recent discoveries have highlighted the need to revisit the classical catalytic cycle and adopt a revised framework that integrates static structural insights with the dynamic interplay between redox components.^23–27^ These discoveries include the widespread occurrence and potential physiological role of O_2_ uncoupling,^23–25^ the successful engineering of hybrid proteins incorporating non-native redox partners,^27^ and the construction of functional fusion proteins linking redox partners directly to the oxygenase domain.^26^

In this perspective, we mainly focus on the structural and mechanistic features of the oxygenase component of RO systems, which underpin the powerful chemical transformations catalyzed by these enzymes and highlight recent advances in the understanding of the complex principles guiding RO reactivity. We begin with examining the quaternary structure, which stabilizes the oligomeric organization required for the proper spatial arrangement of the metal cofactors and, consequently, the catalytic performance. Next, we summarize how the local environment surrounding the Rieske cluster modulates its redox potential and how the catalytic domain contributes to fine-tuning the reactivity at the active site. We then discuss the electron transfer mechanisms, highlighting the dynamic and selective interactions between oxygenases and their redox partners. Finally, we discuss the current limitations in structural and mechanistic understanding and how engineering strategies, particularly redox partner optimization, could expand the biocatalytic applications of ROs.

## The Quaternary Structure of the Terminal Oxygenase of Rieske Oxygenases

ROs exhibit great diversity in their quaternary structures, largely driven by the catalytic and structural demands of the terminal oxygenase component, rather than the nature of the associated electron transfer partners.^28^ Across structurally characterized systems, these enzymes consistently assemble into C_3_-symmetric oligomers, most commonly taking the form of α_3_ trimers,^13,29–35^ α_3_β_3_ or α_3_α’_3_ heterohexamers,^36–50^ or α_3_α_3_ homohexamers (Table 1).^51^

**Table 1:** Summary of Available Structures of ROs

RO	Organism	Subunit compositions	PDB	Ref.
DMO	*Stenotrophomonas maltophilia*	α_3_	3GKE	^29^
CntA	*Acinetobacter baumannii*	α_3_	6Y8J	^13^
CARDO	*Janthinobacterium* sp. strain J3	α_3_	1WW9	^31^
OMO	*P. putida* strain 86	α_3_	1Z01	^30^
KshA	*Mycobacterium tuberculosis*	α_3_	2ZYL	^32^
SxtT	*Microseira wollei*	α_3_	6WN3	^33^
GxtA	*M. wollei*	α_3_	6WNC	^33^
Stc2	*Sinorhizobium meliloti* 1021	α_3_	3VCP	^34^
GdmA	*Rhizorhabdus wittichii* RW1	α_3_	7QWT	^35^
NDO	*Pseudomonas* sp. strain NCIB 9816-4	α_3_β_3_	1NDO	^44^
CDO	*Pseudomonas fluorescens* IP01	α_3_β_3_	1WQL	^40^
BPDO	*Rhodococcus* sp. strain RHA1	α_3_β_3_	1ULI	^38^
BPDO	*Comamonas testosteroni* sp. strain B-356	α_3_β_3_	3GZY	^37^
BPDO	*Paraburkholderia xenovorans* LB400	α_3_β_3_	2XR8	^48^
NagGH	*Ralstonia* sp. strain U2	α_3_β_3_	7C8Z	^41^
NBDO	*Comamonas* sp. strain JS765 P.	α_3_β_3_	2BMO	^42^
TDO	*P. putida* strain F1	α_3_β_3_	3EN1	^39^
PAHDO	*Sphingomonas* sp. CHY-1	α_3_β_3_	2CKF	^45^
3NTDO	*Diaphorobacter* sp. DS2	α_3_β_3_	5XBP	^36^
TPDO	*C. testosteroni* KF-1	α_3_β_3_	7VJU	^46^
TPADO	*Comamonas* sp. strain E6	α_3_β_3_	7Q04	^47^
IadDE	*Variovorax paradoxus*	α_3_β_3_	8H2T	^49^
HcaEF	*Escherichia coli* K-12	α_3_β_3_	8K0A	^50^
NdmA/NdmB	*P. putida* CBB5	α_3_α’_3_	6ICK/6ICL	^43^
PDO	*Comamonas testosteroni* KF-1	α_3_α_3_	7FJL	^51^

Each α-subunit is composed of two domains: a Rieske [2Fe–2S] cluster domain and a catalytic domain containing a mononuclear iron site (Figure 2a).^1^ In the assembled complex, the spatial arrangement of subunits positions the Rieske cluster of one monomer approximately 12 Å from the catalytic iron center of an adjacent subunit (Figure 2b).^1^ This close proximity enables efficient intersubunit electron transfer, which is significantly shorter than the 45 Å separation that would be required for intrasubunit transfer.^1^ Notably, the Rieske domain is thought to have evolved from a ferredoxin ancestor,^52^ but includes a characteristic insertion that facilitates intersubunit contact with the catalytic domain of an adjacent α-subunit.^44^ Likewise, the extension region on the other side of the Rieske cluster is involved in interactions with a β-subunit.^44^ However, in homohexameric assemblies such as phthalate dioxygenase (PDO), the α_3_α_3_ architecture includes an extended region in the catalytic domain that mediates trimer stacking through specific hydrogen bonds and salt bridges to further stabilize the hexameric structure.^51^

**Figure 2:**
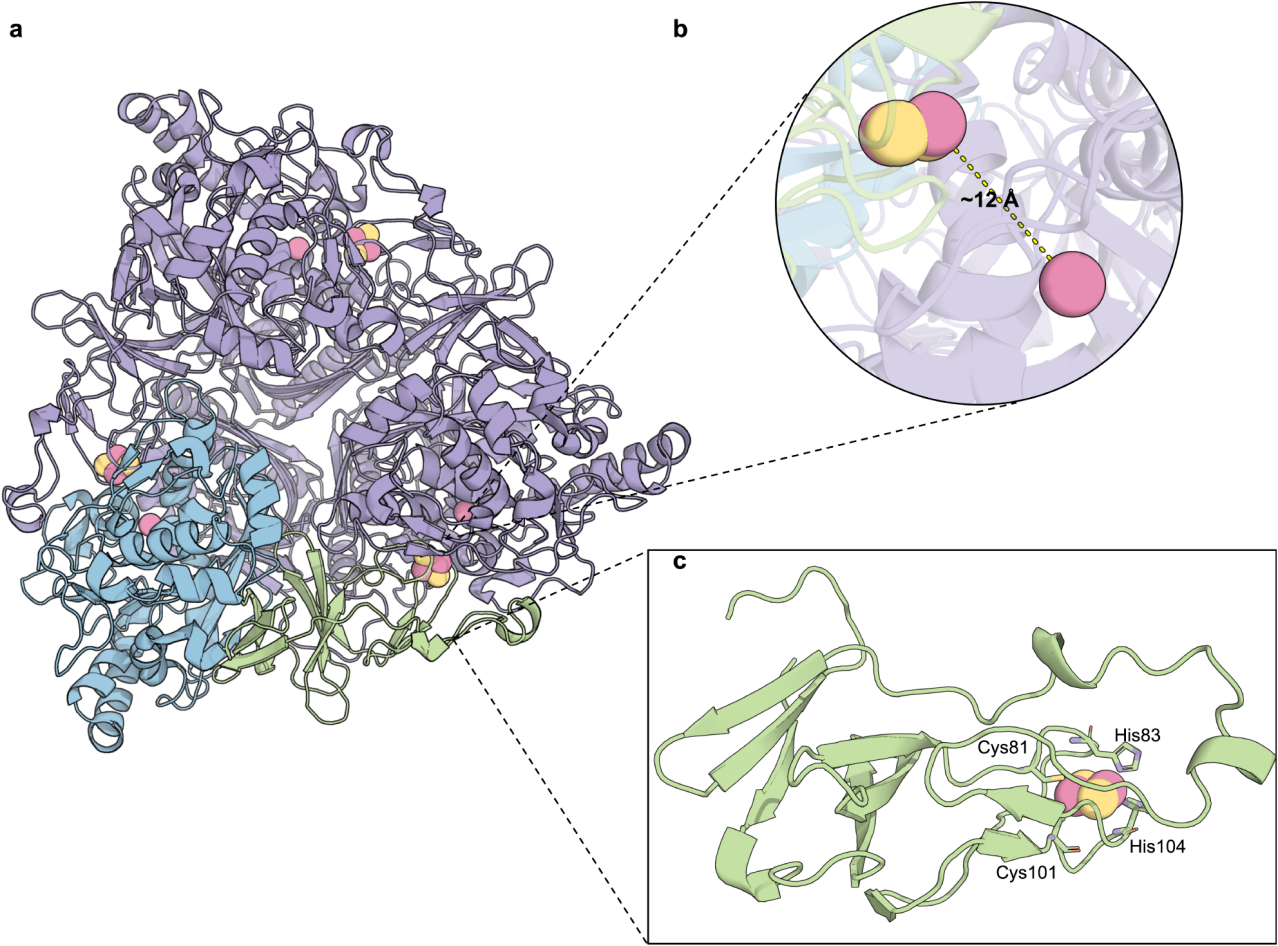
Structure of ROs, exemplified for naphthalene dioxygenase from *Pseudomonas putida* (PDB: 1NDO).^44^ (a) Each α-subunit in ROs contains a Rieske [2Fe–2S] cluster domain (colored in green) and a catalytic domain (colored in blue). (b) The quaternary structure of ROs allows the electron transfer between the Rieske cluster and the mononuclear iron of a separate subunit. (c) Structure of the Rieske domain. The Rieske cluster is coordinated by the side chain of residues Cys81, His83, Cys101 and His104.

The β-subunits, while lacking catalytic centers, play a supportive yet critical role in complex stability and function. They contribute to the structural integrity of the hexameric assembly,^42,44^ facilitate electrostatic or hydrophobic interactions with redox partner proteins,^53^ and enhance the enzyme’s adaptability to a broad range of aromatic substrates.^28^ The N-terminal residues of β-subunits participate in trimer–trimer interactions, while central loop regions mediate both α-β and β-β contacts.^44^ In certain ROs, β-subunits may also contribute to substrate selectivity,^54,55^ though the exact mechanisms remain under investigation.

## The Surrounding Milieu of the Rieske Cluster Contributes to Redox Properties

The surrounding milieu refers to the residues and structural elements near the [2Fe–2S] Rieske cluster that influence its redox properties through electrostatic or hydrogen-bonding interactions. These elements are the primary and secondary coordination spheres. They are essential for the cluster’s redox properties and, thus, its catalytic role in specific oxidative transformations. The Rieske domain adopts a fold primarily composed of antiparallel β-sheets, interspersed with variable numbers of α-helices (Figure 2c). Central to its function is the unique coordination environment of the [2Fe–2S] cluster, which is ligated by residues from two distinct loops within the protein. Each loop contributes one cysteine and one histidine residue, forming the typical Cys-X-His-X(15–47)-Cys-X(2)-His coordination motif of Rieske proteins.^52^

Unlike classical [2Fe–2S] clusters coordinated exclusively by four cysteines,^56^ the Rieske cluster features a distinct coordination scheme. One iron ion (Fe_1_) is coordinated by the thiolate side chains of two cysteine residues, while the second iron ion (Fe_2_) is ligated by the ε-nitrogen atoms of two histidine imidazole rings.^57^ These histidine residues are deeply buried within the interface formed by α- and β-subunits, creating a unique microenvironment.^44^ This asymmetric coordination contributes to a higher redox potential compared to plant-type iron–sulfur clusters, as histidine, being a softer ligand than cysteine, donates less electron density to the metal center.^58^ The cluster features two bridging sulfide ions that form the core of its flat, rhombic structure, together with the iron atoms. In terms of electron transfer, Fe_1_ remains in a ferric state throughout the redox cycle, while Fe_2_ cycles between ferric and ferrous states.^59^

While primary coordination to metal ions governs the fundamental redox properties, the secondary coordination sphere also plays a significant role in tuning the redox potential, especially among ROs that share similar primary structures. Studies on Rieske-type ferredoxins have shown that the stepwise addition of ionizable residues near the cluster can progressively increase the redox potential.^60^ This principle underlies the distinction between high-potential Rieske proteins found in respiratory electron transport chains and the lower-potential Rieske-type ferredoxins found in bacterial dioxygenase systems.^60^ One notable structural feature in high-potential Rieske proteins is the presence of a disulfide bond between two cysteine residues located approximately 5–5.5 Å from the Rieske cluster.^61,62^ This bond links the two cluster-binding loops and partially encloses the cluster, leading to a substantial reorientation of nearby peptide segments.^62^ This rearrangement removes two amide protons from proximity to one of the sulfide atoms (S_2_) in the cluster and repositions the dipoles of nearby peptide bonds away from the cluster, thereby modulating the electrostatic environment.^62^ Additionally, this disulfide bridge affects interactions on the opposite face of the cluster, specifically influencing hydrogen bonding between a backbone amide and the sulfide atom adjacent to the histidine ligand.^63^ Other residues within the secondary coordination sphere also influence the redox behavior. For instance, in the high-potential bc_1_ complex Rieske protein (bc1R), Ser163 and Tyr165 form hydrogen bonds with the cluster sulfide atom (S_1_) and S_γ_ of the first cysteinyl ligand, respectively.^63^ These interactions stabilize the reduced form by decreasing the electron density on the sulfur ligands. Serine is rare at the corresponding position in ROs, based on the sequence alignment of 44 representative RO sequences, while the conserved tyrosine position is variably substituted with phenylalanine, tryptophan, or, in the unique case of PDO, methionine.^64^

Beyond these specific positions, additional conserved charged and aromatic residues are observed near the Rieske cluster in most ROs, such as arginine residues located both before and after the first histidine.^64^ While their exact functions remain to be elucidated, they are likely involved in fine-tuning the protein’s redox behavior. Factors such as hydrophobicity, net charge, and π-stacking interactions are known to modulate electron transfer rates by minimizing the reorganization energy or by selectively stabilizing particular redox states.

Overall, the redox potential of the Rieske cluster in ROs arises from a complex interplay between the redox-active Fe_2_ site and its ability to delocalize electron density through both its immediate ligands and the surrounding protein environment. This finely balanced system enables ROs to achieve the precise redox characteristics necessary for their catalytic roles in specific oxidative transformations.

## Structural Determinants of Reactivity in Rieske Oxygenases

The catalytic diversity of ROs arises from precise structural adaptations within their catalytic domains that collectively orchestrate substrate recognition, oxygen activation, and reaction selectivity. While the Rieske cluster responsible for electron transfer is largely conserved across the RO family, the catalytic domain displays notable sequence and structural variability. These differences represent evolutionary adaptations that enable distinct substrate specificities and regio-, stereo-, and chemoselective oxygenation chemistries across the RO repertoire.^28^

The architecture of the substrate-binding pocket contributes to substrate recognition by influencing how substrates are oriented relative to the mononuclear iron and thus influences the nature and outcome of oxygenation. Most structurally characterized ROs act on hydrophobic aromatic substrates and exhibit active sites enriched in hydrophobic and aromatic residues, which facilitate substrate stabilization through π–π stacking and van der Waals interactions. For example, biphenyl dioxygenase (BPDO) and PDO contain aromatic residues such as Phe227, Phe277, Phe376, and Phe382 (in BPDO), and Phe280, and Phe339 (in PDO), which help to orient and retain the substrate within the active site for efficient catalysis (Figure 3a,b).^37,51^ Similarly, in angular dioxygenases like carbazole dioxygenase (CARDO), the enzyme targets angular positions near heteroatoms, which assist in substrate stabilization through hydrogen bonding (Figure 3c).^65,66^ Terephthalate dioxygenase (TPADO) leverages polar residues to anchor its terephthalate substrate via salt bridges with carboxylate groups, ensuring proper orientation (Figure 3d).^46^ Nitrobenzene dioxygenase (NBDO) provides another example, where Asn258 forms a hydrogen bond with the oxygen atom of the nitro group, guiding regioselective attack (Figure 3e).^42^

**Figure 3:**
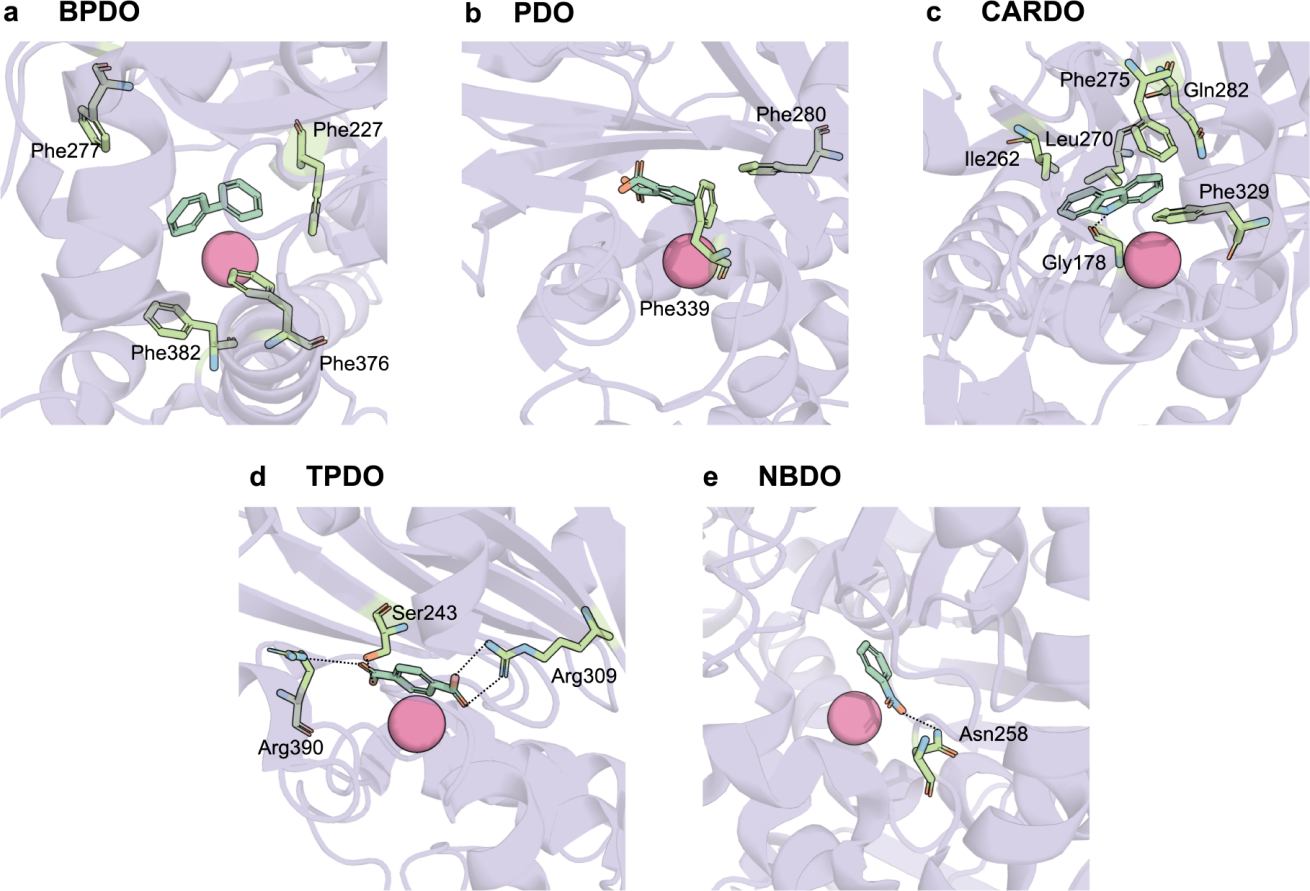
The architecture of the substrate-binding pocket governs substrate positioning in ROs. Substrate-binding pockets are exemplified for five representative RO systems. (a) Residues Phe227, Phe277, Phe376, and Phe382 in biphenyl dioxygenase (BPDO) are important for π–π interactions with biphenyl (PDB: 3GZX).^37^ (b) Residues Phe280 and Phe339 in phthalate dioxygenase (PDO) form π–π stacking interactions with the aromatic ring of phthalate (PDB: 7 V25).^51^ (c) The hydrogen-bond interaction between the imino nitrogen of carbazole’s middle ring and the carbonyl oxygen of Gly178 in carbazole dioxygenase (CARDO) is critical for substrate binding orientation (PDB: 2DE7).^65^ (d) Residue R309 in terephthalate dioxygenase (TPADO) forms a salt bridge with one of the carboxylate groups of terephthalate, while Ser243 forms a hydrogen bond with the second carboxylate group (PDB: 7Q05).^47^ (e) Asn258 in nitrobenzene dioxygenase (NBDO) can form a hydrogen bond with the nitro group of the nitroarene substrates (PDB: 2BMQ).^42^

These specific protein–substrate interactions suggest a broader principle: active-site residues govern substrate alignment through defined steric and electrostatic interactions. Extending this logic, it is plausible that in enzymes like benzoate dioxygenase, anthranilate dioxygenase, and aniline dioxygenase, where vicinal dioxygenation is observed, similar polar or hydrogen-bonding interactions between functional groups—carboxylate or amino—and the enzyme scaffold may play a guiding role in substrate positioning.

Beyond positioning, the active site also exerts stereochemical control. In NDO, for example, the residue Phe352 defines a chiral pocket that enforces *cis*-dihydroxylation.^67,68^ Substitutions at this position influence the product configuration while maintaining regioselectivity.^69–71^ Moreover, in toluene dioxygenase (TDO), replacing the corresponding residue Phe366 with valine inverts the enantioselectivity of naphthalene (**19**) oxidation (Scheme 1), illustrating how subtle alterations can reshape the stereochemical outcome.^72^

**Scheme 1:**
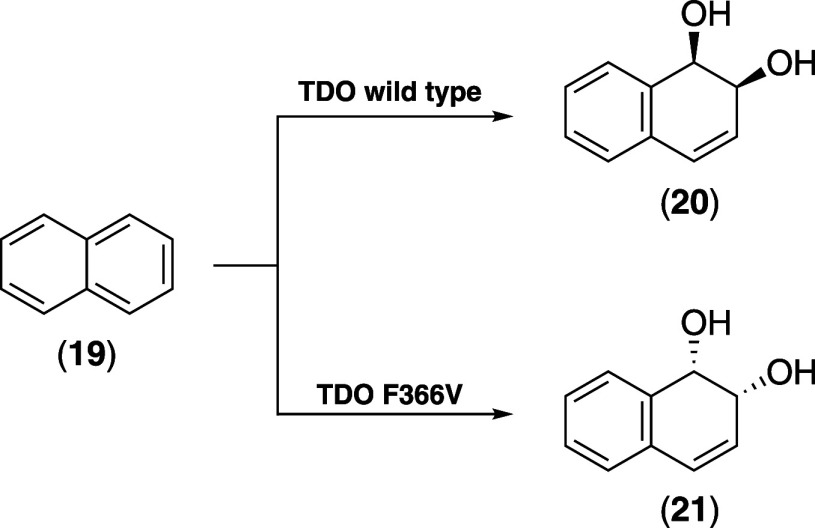
Engineering the Active Site of ROs Can Alter Their Enantioselectivity^a^

a The TDO variant F366V enables a switch in enantioselectivity from (+)-(1*R*,2*S*)-**20** to (−)-(1*S*,2*R*)-**21** with an *ee* of 90% in the dihydroxylation of naphthalene (**19**).

A compelling conceptual framework that synthesizes insights from active site engineering is the “ruler model,” originally proposed based on structural studies of the RO TsaM.^73^ This model posits that the vertical positioning of the substrate relative to the mononuclear iron center dictates the type of oxygenation reaction that occurs: substrates positioned closer to the iron favor dioxygenation, those at an intermediate distance undergo monooxygenation, and higher placements lead to sequential monooxygenation.^73^

This spatial dependence is consistent with findings in other systems. For example, in *N*-demethylase A and B (NdmA and NdmB), the distance between the *N*-methyl group and the iron center is a critical determinant for regiospecific *N*-demethylation.^43^ The ruler model thus provides a unifying spatial framework to describe the catalytic diversity of ROs and serves as a practical guide for enzyme engineering aimed at tuning reaction outcomes.

Structural flexibility also plays a critical role in determining reactivity. Loop regions at the entrance to the active site act as dynamic gates that regulate substrate access and product release. Structural analysis of CARDO supports this mechanism. In the presence of the substrate, loop regions Leu202–Thr214 and Asp229–Val238 were observed to shift toward the substrate-binding pocket (Figure 4a).^65^ These changes, including a rigid-body motion and flipping of key residues like Ile231 and Phe204, form a lid-like structure that encloses the substrate, stabilizes binding, and likely prevents solvent intrusion.^65^ Such dynamic gating not only aids in efficient catalysis but also helps define the enzyme’s substrate specificity and the regioselectivity of oxidation. Notably, these loops vary considerably in length and conformation among the ROs. Sequence alignments and structural modeling, based on the crystal structure of 3-ketosteroid 9α-hydroxylase (KshA), identified a 58-amino-acid β-sheet region within the helix-grip fold as a variable segment influencing substrate binding (Figure 4b).^74^ Chimeric swapping of this region between KshA1 and KshA5 homologues generated functional enzymes with altered substrate preferences, highlighting how sequence variability outside the canonical active site can modulate function.^74^ Further dissection of the β-sheet region revealed that a highly variable loop at the entrance of the active site significantly contributes to substrate specificity.^74^ This loop may undergo conformational changes, guiding substrate entry, stabilizing binding, and influencing the reaction outcome. Similar functional plasticity has been demonstrated in RO SxtT (Figure 4c). Mutating the loop residue Arg204 to lysine, the corresponding residue in the RO GxtA, partially redirected hydroxylation to the C11 position favored by GxtA.^75^

**Figure 4:**
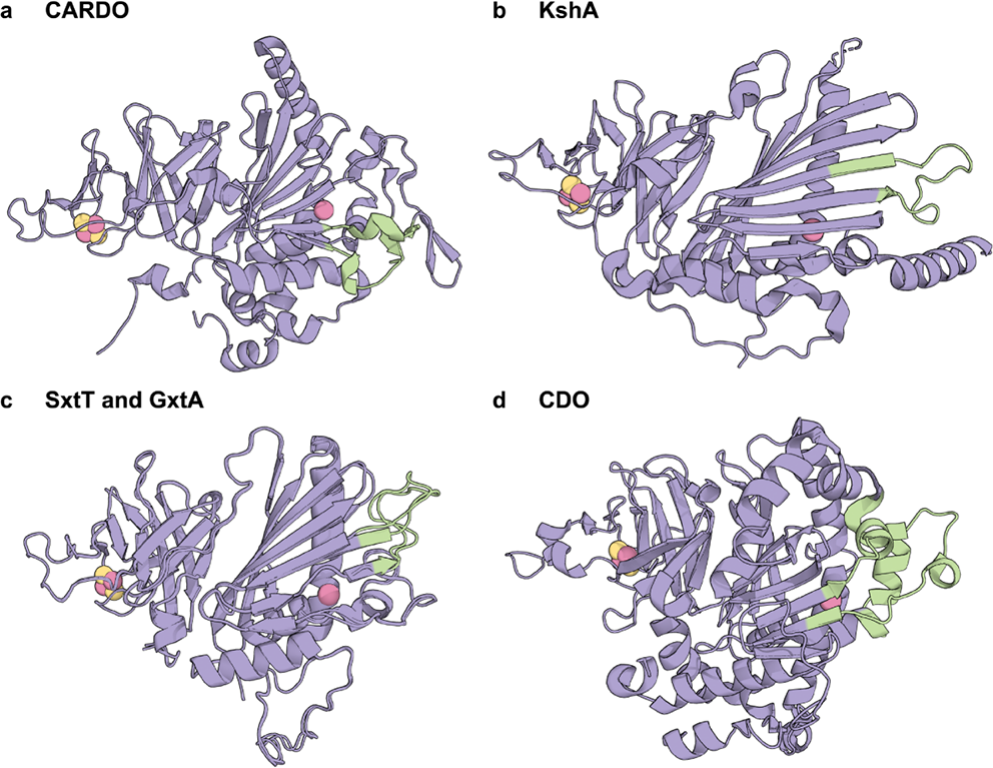
Loop regions involved in modulating RO reactivity are highlighted in green in (a) CARDO, (b) KshA, (c) SxtT and GxtA, and (d) CDO.

Recent loop engineering studies on CDO demonstrated that deletion or insertion of linkers in the loops above the active site altered substrate specificity (Figure 4d).^76^ Deletion enhanced activity toward bulkier substrates due to an expanded entrance to the active site or the creation of a new entrance route, while loop extension dramatically inverted regioselectivity, likely by modifying substrate positioning during entry.^76^

Substrate access tunnels provide another layer of control. Because the catalytic site is typically buried to protect reactive intermediates from the solvent, substrates must traverse a defined access route. These tunnels are not static and adjust in shape and chemical character to accommodate diverse substrates. Structural and computational studies of NDO identified four residues (Phe224, Leu227, Pro234, and Leu235) forming a narrow bottleneck that governs substrate entry.^77^ Notably, Phe224 serves a dual role, both modulating access via π–π interactions and stabilizing the bound substrate in a catalytically competent pose.^77^ These features exemplify how the chemical character and spatial arrangement of tunnel-lining residues can define the substrate scope of the ROs. Both comparative analysis of related enzymes SxtT and GxtA and structure-guided engineering of TsaM have shown that even minor differences in tunnel architecture can shift hydroxylation site selectivity, further establishing the tunnel as a functional extension of the active site.^33,75,78^

Although β-subunits do not directly participate in catalysis or tunnel formation, they can probably exert allosteric control over enzyme activity. In BPDO, swapping β-subunits modulated substrate preferences between biphenyl and toluene,^79^ likely by influencing loop dynamics or substrate positioning through intersubunit contacts.

Overall, the reactivity and selectivity of ROs are governed by a complex interplay of structural features within their catalytic domains. Key determinants, including the shape and chemistry of the active site, the dynamics of loop regions, the architecture of substrate access tunnels, and interactions at the subunit interfaces, collectively create an adaptable catalytic framework. Emerging studies reveal recurring structural motifs that help to rationalize catalytic outcomes across the RO family. For instance, surface loop regions adjacent to the active site not only control the accessibility of substrates but also participate in substrate recognition and proper accommodation.^43,74–76^ The substrate access tunnels further regulate catalytic specificity, with their shape, hydrophobicity, and electrostatic gradients forming selective filters that guide substrates toward the active site.^75,77,78^ Furthermore, catalytic outcomes can be modulated by adjusting the spatial constraints at the top of the active site, enabling switching between dioxygenation, monooxygenation, and sequential monooxygenation reactions.^73^

Despite these advances, the precise mechanisms by which more distal residues and long-range structural elements modulate the active site reactivity remain poorly understood. Elucidating these influences will be critical for explaining the full range of catalytic behavior observed across the RO family. Continued investigation into these structural principles will not only refine our understanding of enzymatic evolution but also guide the rational design of ROs with improved performance for biocatalytic and environmental applications.

## O_2_ Uncoupling and Its Implications for Reactivity and Adaptability

In ROs, oxygen uncoupling refers to the incomplete coupling between the activation of molecular oxygen and the hydroxylation of the substrate. This process leads to the formation of reactive oxygen species (ROS), such as hydrogen peroxide, instead of the desired product. It reflects a loss of electron economy and can impact catalytic efficiency. Rather than being a rare occurrence, oxygen uncoupling is prevalent across many ROs and is increasingly recognized as a fundamental aspect of their catalytic behavior.

Recent studies have shown that substrate-specific oxygen uncoupling is influenced by how the substrate affects the geometry and electronic environment of the active site.^23,24^ Substrates can modulate the coupling efficiency not only through their binding orientation but also by affecting the timing and energetics of electron transfer.^23,24^ Although the substrate does not coordinate directly to the nonheme iron center, it can alter the positioning of active site residues and thus influence the access of oxygen to the iron.^80^ This phenomenon of oxygen uncoupling is also proposed to play a role in the evolutionary adaptation of ROs.^25^ In microbial environments, exposure to persistent oxidative stress can create selective pressure favoring enzyme variants that reduce uncoupling.^25^ Structural adaptations, such as modified substrate tunnels or altered oxygen access channels, have been associated with increased coupling efficiency.^25^ Such modifications may enhance microbial fitness when metabolizing challenging or newly encountered substrates,^25^ indicating that uncoupling is not only a biochemical limitation but also a factor in evolutionary adaptation.

Building on insights from natural adaptations that reduce oxygen uncoupling within the oxygenase component, recent efforts have begun exploring how modifying the associated redox partners impacts overall catalytic efficiency. The reductase and ferredoxin are crucial for ensuring timely and coordinated electron transfer; inefficiencies at this level can contribute to ROS formation. These ROS not only represent wasted reducing equivalents but also can damage the enzyme itself, ultimately impairing catalytic turnover. In response, hybrid CDO systems have been developed that introduce non-native redox partners with improved electron transfer characteristics. These systems have led to a measurable reduction in ROS formation.^27^ This example demonstrates that tailoring the entire electron transfer chain, not just the oxygenase, can be a powerful strategy for improving the catalytic performance.

## Electron Transfer Driven by Redox Partner Recognition

Electron transfer that is well-timed and tightly coupled to substrate oxidation is critical for sustaining the catalytic activity of ROs. Their redox partners—typically a shuttle protein, ferredoxin, in three-component systems or a reductase in two-component systems^1^—must form structurally compatible and energetically favorable complexes with the terminal oxygenase to support catalysis while minimizing deleterious side reactions.

The structural basis of the redox partner interaction has been elucidated in a few model systems. A notable example is the complex formed by NdmA and the plant-type ferredoxin domain of NdmD, which is involved in caffeine oxidative *N*-demethylation. NdmD, a multidomain reductase, transports electrons from NADH to the terminal oxygenase NdmA or NdmB.^81^ The crystal structure reveals that one molecule of the C-terminal plant-type ferredoxin domain of NdmD engages with two NdmA subunits through a hydrophobic interface at the trimer boundary, highlighting how spatial positioning can be optimized within hetero-oligomeric complexes to promote sequential catalysis.^43^

In addition, the reductase usually exhibits moderate promiscuity in redox partner compatibility. For example, terminal oxygenases from two-component systems can often function with non-native reductases if redox potential alignment permits effective electron transfer.^33,73^ In such cases, thermodynamics, rather than precise structural matching, may govern electron flow. This flexibility has enabled engineering of hybrid systems, where substituting native reductases with alternatives improves catalytic activity.^27^

In contrast, interactions between ferredoxin and oxygenase are typically more selective. Structural studies of the complex formed between ferredoxin from *Pseudomonas resinovorans* CA10 and CARDO oxygenase from *Janthinobacterium* sp. J3 reveal that three ferredoxin molecules bind to the oxygenase trimer, with each ferredoxin positioned at the interface between two adjacent oxygenase subunits.^65^ This arrangement is stabilized through conformational changes in both proteins. Specifically, movements of side chains such as Trp15 and Val351 in the oxygenase, along with Phe67 and Pro83 in the ferredoxin, facilitate the formation of hydrophobic interactions.^65^ Notably, redox state-dependent conformational changes, such as the repositioning of the ferredoxin Phe67 side chain away from the Rieske cluster, appear to play a critical role in modulating the association and dissociation of the oxygenase and ferredoxin complex.^65^ Similar redox-sensitive interactions have been observed in BPDO reductase and ferredoxin, emphasizing the importance of dynamic conformational regulation in high-affinity binding and functional electron transfer.^82^

Comparative structural analyses across classes III and IIB of CARDO further suggest the diversity in redox partner interaction modes. While conserved residues maintain core redox contacts, variations in nonconserved residues define interaction geometry and electron transfer specificity.^83^ Recent studies combining docking simulations and mutational analysis of CDO suggest that the ferredoxin binding site is located in a side-wise depression formed at the interface between the α- and β-subunits, differing from the top-wise site on α3-type oxygenase.^53^ These findings highlight the structural plasticity of ROs in accommodating redox partners through class-specific recognition surfaces.

Electron transfer between the Rieske [2Fe–2S] cluster and the mononuclear iron center in ROs is widely believed to proceed via a proton-coupled electron transfer (PCET) mechanism, mediated by a highly conserved aspartate residue.^84^ This aspartate forms a hydrogen-bonding bridge that links the mononuclear iron site of one subunit to the Rieske cluster of a neighboring subunit, engaging specifically with the ligand histidines coordinating both metal centers.^1^ This intersubunit connectivity is thought to facilitate efficient intracomplex electron flow during catalysis. However, the electron transfer pathway from the ferredoxin Rieske cluster to the oxygenase Rieske cluster remains experimentally unvalidated. Computational predictions of this interprotein electron transfer pathway have yielded inconsistent results, with significant variability in the identity and positioning of the residues involved (Figure 5).^36,53,65,83^ Notably, unlike the aromatic amino acid-mediated electron hopping pathways observed in other redox enzymes, such as tryptophan and tyrosine chains in DNA photolyase or ribonucleotide reductase, which support multistep electron transfer via discrete redox-active π-systems, the predicted routes in ROs typically involve noncanonical mechanisms.^85–88^ These often rely on electron tunneling through main-chain peptide bonds or networks of hydrogen bonds, where the electron is transferred directly from donor to acceptor via orbital superexchange without occupying intermediate atomic orbitals.

**Figure 5:**
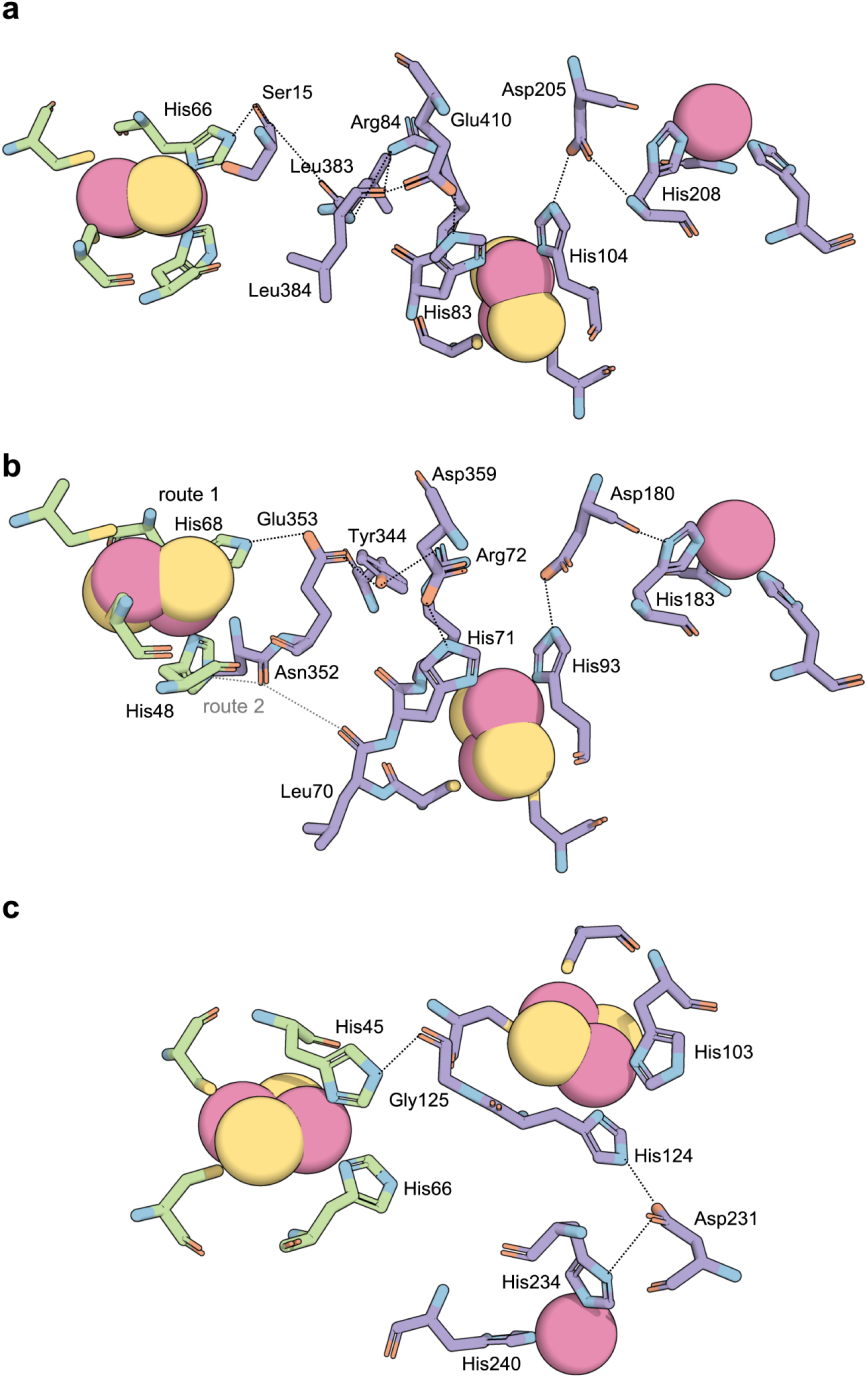
Predicted electron transfer pathway from the Rieske cluster of ferredoxin to the nonheme iron of oxygenase in (a) NDO,^36^ (b) CARDO,^65^ and (c) CDO.^53^ Residues in ferredoxin and oxygenase are colored light green and lilac, respectively. The hydrogen bonds that mediate electron transfer are indicated by black dashed lines.

The structural complementarity between ROs and their redox partners, including geometric alignment and specific protein–protein interactions, plays a critical role in modulating the dynamics of electron transfer. Optimizing this structural interface offers opportunities to enhance RO catalytic efficiency. Covalent fusion of redox partners, for example, can improve electron transfer efficiency by ensuring constant proximity and orientation, thus minimizing electron loss or ROS formation.^26^ At the same time, engineered compatibility between modular components offers the potential to create customizable electron transfer chains for synthetic biology or industrial biocatalysis.

## Discussion and Future Directions

ROs serve as powerful models for understanding nature’s orchestration of electron transfer and oxidative chemistry, while also offering potential as versatile biocatalysts. However, a significant limitation in the current research landscape is the lack of structural data. Although over 20 crystal structures have been resolved, this represents only a small fraction of the vast and phylogenetically diverse family of ROs. In particular, high-resolution structures of complexes with redox partners, substrate- or product-bound forms, and engineered variants that reveal critical interdomain interactions remain rare, and these functionally important conformations are not reliably captured by predictive tools such as AlphaFold3. One major reason is the difficulty in the heterologous expression of these enzymes. Many ROs originate from nonmodel bacteria and are large, multisubunit enzymes that are dependent on proper metal cofactor incorporation for stability and function. In heterologous hosts such as *E. coli*, inefficient folding and limited availability of Fe–S clusters often result in misfolded or inactive proteins.^89^ This impedes both structural characterization and mechanistic studies and may obscure unique features, such as the dual electron transfer pathway recently identified in aminopyrrolnitrin oxygenase (PrnD).^89^ Addressing this bottleneck will require improved expression systems, such as engineered bacterial strains with enhanced cofactor assembly pathways.^89^ Additionally, advances in cryo-EM may offer a promising alternative for structural characterization of ROs that are refractory to crystallization, while also enabling the study of their dynamic behavior as well as interdomain interactions between redox partners. In particular, structural characterizations of redox partner interactions with the terminal oxygenase would be highly desirable to shed light on the intricate electrostatic and hydrophobic interactions that guide protein–protein interactions and, consequently, efficient electron transfer. Elucidating the residues involved in binding and productive electron transfer to the Rieske cluster within the oxygenase, and further to the nonheme iron center, could provide experimental evidence of the electron transfer pathways adopted by oxygenases. This might provide a starting point for modulating electron transfer efficiency and coupling rates by engineering an efficient, continuous supply of electrons from the redox partner to the enzyme’s active site to achieve higher catalytic activity. Ultimately, this could lead to improved catalytic efficiency of ROs and facilitate their use in the large-scale biocatalytic synthesis of active pharmaceutical ingredients.

A second critical limitation lies in our incomplete understanding of the catalytic mechanism, especially the oxygen activation and substrate functionalization steps. While substrate binding has been structurally characterized in some ROs, mechanistic insight into O_2_ activation and subsequent C–H hydroxylation or other oxidative transformations remains elusive.^90^ This gap largely stems from the transient nature of reactive species, which hinders direct observation of intermediates in action.^80^ Moreover, structural data on ROs performing atypical transformations, such as desaturation and oxidative ring cleavage, are nearly nonexistent. Consequently, we lack a unified framework to explain how ROs achieve diverse catalytic specificity from a common mechanistic scaffold. This challenge is further complicated by our limited understanding of how the β-subunit contributes to substrate recognition and positioning, despite its known role in influencing specificity in certain ROs.^79,91^

A lack of knowledge regarding the specificity between ROs and their redox partners poses a significant barrier to the functional characterization of novel ROs. This challenge is particularly pronounced for ROs involved in natural product biosynthetic pathways, where native redox partners are frequently not colocalized with oxygenase genes and often remain unidentified. Without clearly defined redox interactions, evaluating enzyme activity or determining how electron transfer efficiency influences catalytic outcomes becomes challenging. Addressing this limitation requires more systematic identification of native redox partners and, more importantly, a deeper understanding of the molecular determinants that govern specificity and compatibility within these multicomponent systems. This knowledge could ultimately support the development of several classes of “universal” redox partners engineered to function broadly with ROs that lack identified or well-characterized redox systems.

Looking ahead, redox partner engineering offers a particularly promising avenue for enhancing the RO performance in both mechanistic studies and applied catalysis. In ferredoxin-dependent ROs, electron transfer must be efficient yet reversible. Tight binding improves electron flow but can limit turnover, while weak interaction reduces electron transfer rates. Balancing this specificity-efficiency trade-off remains a key challenge. Moreover, emerging evidence suggests that redox partners may also play regulatory roles,^92,93^ influencing conformational dynamics or catalytic timing. Drawing from recent advances in cytochrome P450 systems, redox partner interface engineering, which targets surface residues to optimize redox center orientation and proximity, has shown success in improving coupling efficiency and reaction rates.^94,95^ These strategies, alongside rational design of electron transfer pathways and manipulation of electron transfer routes via mutagenesis or cofactor modulation,^96,97^ are prime strategies for application to ROs. Additionally, recent advances in cytochrome P450 systems could inspire artificial fusion approaches of redox partners or the generation of self-sufficient ROs.^98–101^ These approaches could further our mechanistic understanding of the underlying electron transfer principles and the generation of more efficient RO systems. Though a successful case study has been recently reported,^26^ further advances are still needed to fully realize the potential of this engineering approach, especially to address the attenuated catalytic activity and coupling efficiency of the fused systems. Although the recent explosion of genomic information has enabled the identification of several naturally self-sufficient P450s,^102^ no self-sufficient RO systems have been identified yet. One could envision developing a ligation-independent cloning vector to generate a library of redox partners fused to a specific oxygenase of interest in order to rapidly screen for an optimal construct. Solving the crystal structures of these fusions might then prove useful for generating structural models and designing artificial fusion constructs more rationally. Exploring these strategies in ROs could significantly enhance their catalytic efficiency and expand their application in natural product synthesis, sustainable production of pharmaceutically and industrially relevant compounds, and bioremediation.

In summary, ROs have great potential for a variety of bioremediation and biocatalysis applications. However, their multidomain nature poses significant challenges for large-scale applications beyond simple biochemical characterization. Creating artificial ROs with noncognate reductase and ferredoxin partners could inspire the engineering of catalytically self-sufficient systems and lead to the discovery of natural ROs that are catalytically self-sufficient. In addition to summarizing recent knowledge of electron transfer properties within the RO family, we have outlined several protein engineering developments that could be adapted to optimize RO systems. Identifying architectural elements inside and outside the RO active site that promote changes in reaction selectivity, broaden substrate scope, or amplify useful promiscuous activity provides important starting points to pinpoint RO engineering “hotspots” and explain how these architectural regions impact the catalytic outcome. Many of these studies suggest that RO reactivity and selectivity are assembled through the careful selection of quaternary architecture and the modification of the active site, substrate entrance tunnel, and flexible protein loops.^75–77,103^ Thus, future protein engineering efforts of ROs warrant attention to structural elements, such as subunit–subunit interactions, entrance tunnels, and flexible loops, to further capitalize on our growing molecular understanding of these promising biocatalysts.
